# Relationship between oxidative stress and lifespan in *Daphnia pulex*

**DOI:** 10.1038/s41598-022-06279-4

**Published:** 2022-02-11

**Authors:** Benedicth Ukhueduan, Charles Schumpert, Eunsuk Kim, Jeffry L. Dudycha, Rekha C. Patel

**Affiliations:** 1 Department of Biological Sciences, University of South Carolina, 700 Sumter Street, Columbia, SC 29208 USA; 2 School of Earth Sciences and Environmental Engineering, Gwangju Institute of Science and Technology, Gwangju, 61005 Korea

**Keywords:** Ageing, Mitochondrial proteins

## Abstract

Macromolecular damage leading to cell, tissue and ultimately organ dysfunction is a major contributor to aging. Intracellular reactive oxygen species (ROS) resulting from normal metabolism cause most damage to macromolecules and the mitochondria play a central role in this process as they are the principle source of ROS. The relationship between naturally occurring variations in the mitochondrial (MT) genomes leading to correspondingly less or more ROS and macromolecular damage that changes the rate of aging associated organismal decline remains relatively unexplored. MT complex I, a component of the electron transport chain (ETC), is a key source of ROS and the *NADH dehydrogenase subunit 5 (*ND5) is a highly conserved core protein of the subunits that constitute the backbone of complex I. Using *Daphnia* as a model organism, we explored if the naturally occurring sequence variations in ND5 correlate with a short or long lifespan. Our results indicate that the short-lived clones have ND5 variants that correlate with reduced complex I activity, increased oxidative damage, and heightened expression of ROS scavenger enzymes. *Daphnia* offers a unique opportunity to investigate the association between inherited variations in components of complex I and ROS generation which affects the rate of aging and lifespan.

## Introduction

Understanding the molecular basis of aging is a priority as age is the single most important risk factor for human pathologies such as cancer, diabetes, cardiovascular disorders, and neurodegenerative diseases^1^. Accumulating evidence supports that aging starts with macromolecular damage leading to cell, tissue and ultimately organ dysfunction^2^. This intrinsic process can be viewed as an inevitable outcome of the cellular biochemistry, and forms a basis from which the diseases of older age originate. The cellular product that causes the most damage is the intracellular reactive oxygen species (ROS)^3^. Mitochondria play a central role in regulating the rate of macromolecular damage, as they are the principle source of ROS^4–6^. So far, the contribution of mitochondria to aging has been studied with a view that mitochondrial DNA (mtDNA) is the primary target of the ROS generated by mitochondria. Thus, somatic mtDNA mutations are reported in normal aging, particularly in post-mitotic tissues such as skeletal muscle and neurons, leading to a corresponding decline in the mitochondrial function with age^7–9^. Although this decline leads to further ROS production, cellular damage, and aging associated cellular dysfunction^10^; such observations do not show a causal relationship between inherited variations in mitochondrial efficiencies and variations of aging. The causal relationship between naturally occurring variations in the mitochondrial genomes leading to differences in the mitochondrial electron transport chain (ETC) efficiencies that could result in correspondingly less or more ROS and macromolecular damage to change the rate of aging associated organismal decline has remained relatively unexplored.

The mitochondrial ETC is comprised of 5 multi-subunit complexes (complex I- complex V), the last one being the ATP synthase^11^. Of the complexes 1–5 of the ETC, complex I (NADH:quinone oxidoreductase) is the first and largest enzyme, the major electron entry point for the respiratory chain^11^, and is a key source of ROS in mitochondria^12^. Mutations in complex I subunits are implicated in human diseases and aging^13^. Of the 44 subunits (human) of the complex I, 38 are encoded by the nuclear genome and 7 by the mtDNA^10^. The 7 mtDNA-encoded hydrophobic subunits, NADH dehydrogenase (ND) 1–6 and 4L (ND1-ND6 and ND4L), are the core subunits that form the major membrane arm of complex I^14,15^. Mutations in ND subunits disrupt either complex I assembly or enzyme activity and cause ~ 20% of cases of isolated complex I deficiency^16^. In humans, variations in ND5 sequence are associated with mitochondrial encephalomyopathy, lactic acidosis, and stroke-like episodes (MELAS)^17^, as well as some symptoms of Leigh's syndrome^18–20^, and Leber's hereditary optic neuropathy (LHON)^21–24^. Many fatal pathological conditions such as Leigh syndrome, leukoencephalopathy, LHON, mitochondrial myopathy, encephalopathy, MELAS, and Parkinson's disease, and cancer are associated with mutations in the ND genes including the ND5 gene^25,26^. ND5 mutations cause mitochondrial dysfunction due to a decrease in complex I efficiency and increased ROS generation^27^. However, very few studies have been done to relate naturally occurring, non-pathogenic ND5 sequence variations to ETC efficiencies, and ROS generation that can affect rate of aging, senescence, and life span.

In this study, we identified naturally occurring ND5 sequence variations in ecologically distinct populations of the freshwater microcrustaceans *Daphnia pulex* and *D. pulicaria* and examined its association with complex I enzyme activity and level of oxidative damage to total and mitochondrial proteins. *D. pulex* and *D. pulicaria* are short- and long-lived ecotypes of the *Daphnia pulex* species complex^28^ and interbreed frequently^29^. Although zooplankton taxonomists use two different species names for the ecotypes, they are not genetically distinct species in the traditional sense of reproductive isolation. In fact, there is substantial gene flow between the ecotypes^30–33^, and thus persistent differences are likely to be associated with adaptive divergence between ecotypes, rather than true species differences that have accumulated due to non-adaptive evolutionary processes. For simplicity and to emphasize the lack of reproductive isolation, we refer to them as *D. pulex* throughout the manuscript. We also investigated the activities of ROS scavenging antioxidant mechanisms in the short- and long-lived *Daphnia*. Our results indicate that lower activity of complex I possibly leading to higher levels of ROS leads to correspondingly high levels of oxidative damage to proteins. Although there is a corresponding increase in activities of ROS scavenging antioxidant mechanisms, the short-lived clones show an imbalance between ROS production and removal thus accumulating damaged proteins and possibly contributing to their shorter lifespan. The results are important because they demonstrate that oxidative stress, which has been shown to be a proximate driver of lifespan and aging in other model organisms^34,35^ also correlates similarly in *Daphnia*, a newer model system for research on aging . Our study demonstrates that *Daphnia* is an excellent model system that offers significant advantages with its unique clonal reproduction, availability of genome manipulation techniques, and naturally existing ecotypes with varied lifespans to understand natural determinants of lifespan.

## Results

### Genetic variation of lifespan and ND5 sequence variations in *D. pulex* clones

**Figure 1:**
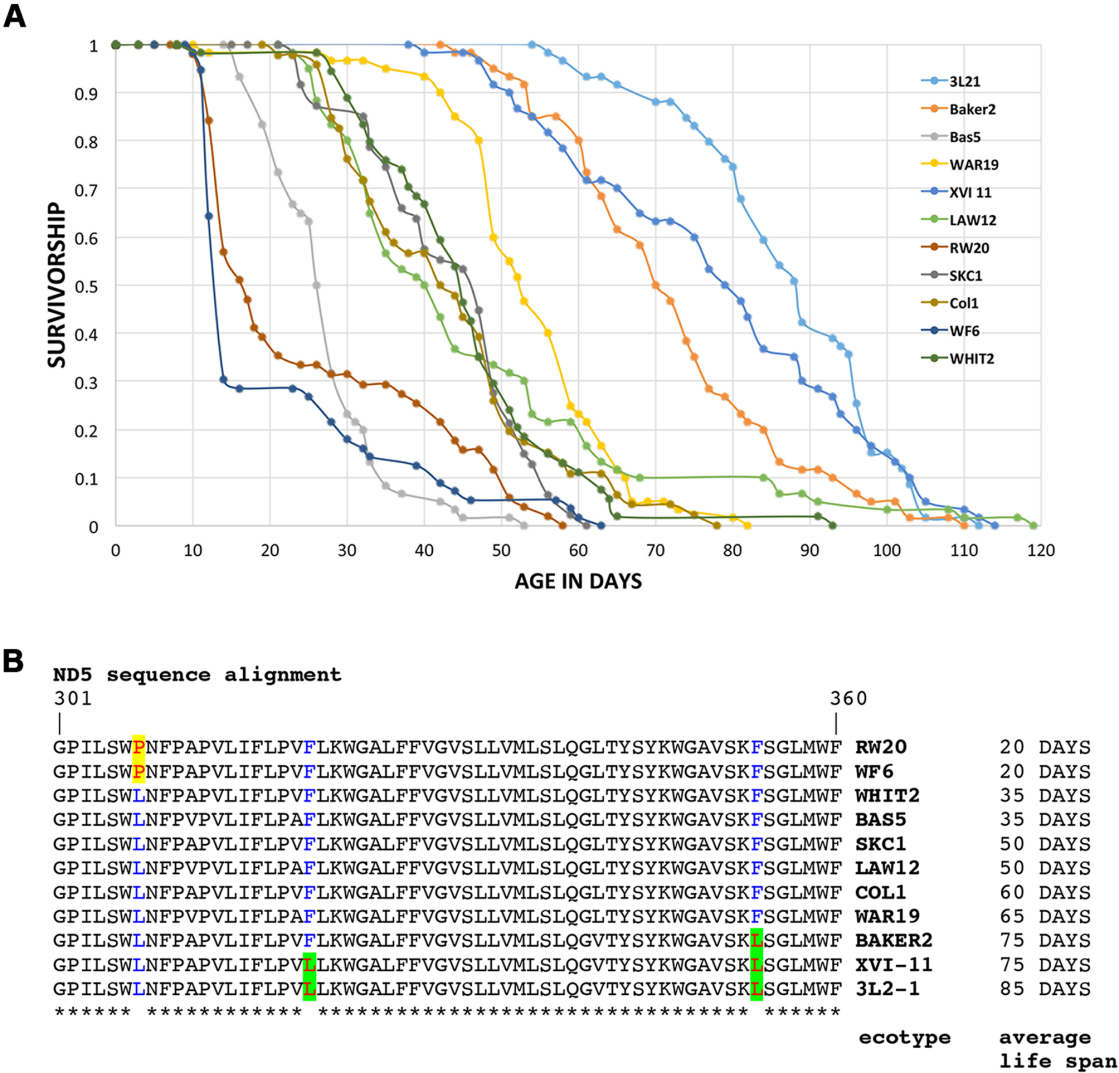
Adult survivorship curves and ND5 sequence alignments. (**A**) Genetic differences of adult survivorship among populations of *D. pulex*. Each line represents a specific clone. Pond clones: RW20, WF6, WHIT2, SKC1, and Col1. Lake clones: Bas5, LAW12, WAR19, Baker, XVI-11, and 3L21. (**B**) ND5 sequence alignments in the order of increasing life spans. Only a short relevant region of the sequence alignments is shown. Red font with yellow highlighting: amino acids specific to the short-lived clones, red font with green highlighting: amino acids specific to the long-lived clones, blue font: positions where amino acid changes are present.

The median lifespans in 11 clones of *D. pulex* were determined by life tables under identical conditions. As seen in Fig. 1A, the median lifespans differ greatly (~ 15 to 85 days) among the clones. Once we had established the survivorship curves, we narrowed our selection of clones for all the future experiments. We chose RW20 and WF6 to represent our short-lived *Daphnia* clones and XVI-11 to represent our long-lived clone. We next investigated whether specific inherited variations of ND5 could be causative of differences in the ETC efficiencies that can then lead to differences in ROS generation and consequently affect the rate of aging and lifespan as observed in other established model organisms for research on aging. ND5 was chosen for initial analysis, as it is used extensively for understanding evolutionary relationships among *Daphnia* populations^31,36^. We sequenced a 1140 bp region of the ND5 gene from the 11 clones to identify specific amino acid variations associated with characteristic short or long lifespans. When we aligned the 11 ND5 protein sequences in the order of increasing life spans (Fig. 1B), a striking pattern emerged. The most significant observations are: (1) the two shortest-lived clones carry a proline at position 307 whereas all other clones have a leucine, (2) the two longest-lived clones carry a leucine at position 320 whereas all other clones carry a phenylalanine, and (3) the three longest lived clones carry a leucine at position 354 whereas all other clones carry a phenylalanine, and (4) these variations are in the conserved C-terminal domain important for ND5 enzymatic activity.

### The MT complex I activity is diminished in the short-lived clones

**Figure 2:**
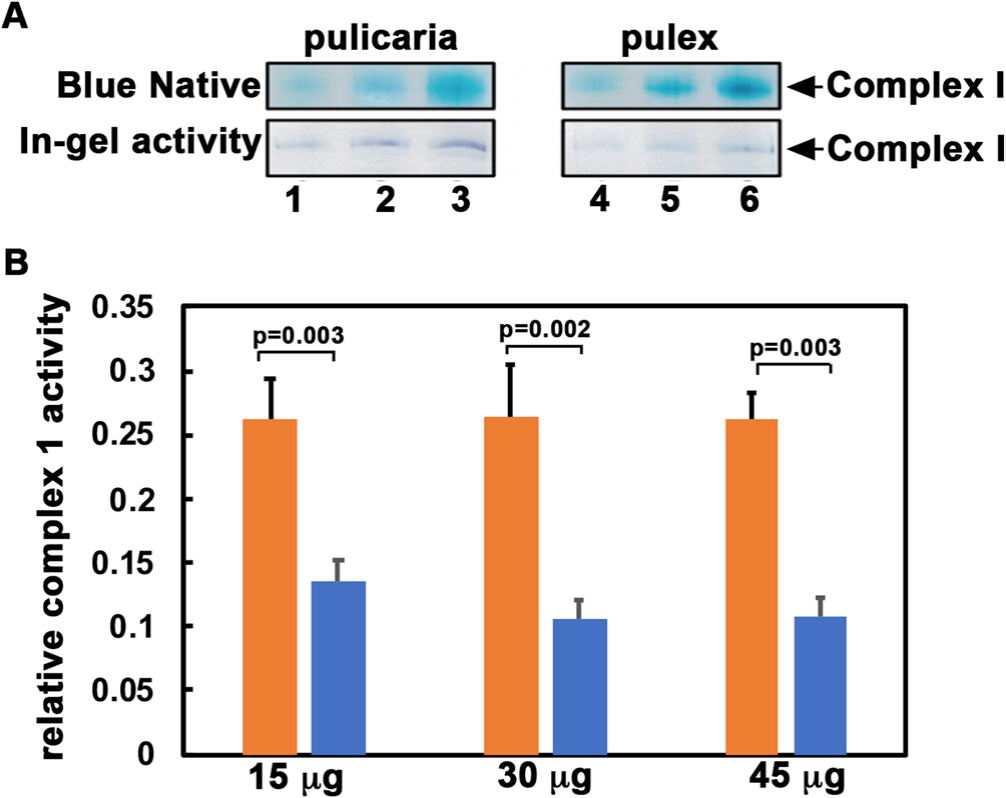
Complex I activity from clones *WF6* and *XVI-11.* (**A**) Blue Native gels and in-gel activity assay for complex I. Extracts made from short-lived WF6 and long-lived XVI-11 clones were run on Blue Native gel for total amount of complex I and a duplicate gel was stained for measuring complex I activity. Lanes 1–3: 15 μg, 30 μg, and 45 μg of total *extract* prepared from XVI-11 mitochondria and lane 4–6: 15 μg, 30 μg, and 45 μg of total extract prepared from WF6 mitochondria. (**B**) Quantification of relative activity of complex I. The band intensities of total complex I and in-gel activity were measured in 4 separate experiments and the complex I activity relative to total amount of complex I was calculated. Blue bars indicate short-lived WF6 samples and the orange bars indicate long-lived XVI-11 samples. The p-values are as indicated.

In order to test if the differences seen in the ND5 sequences correlate with the enzymatic activity of complex I, we compared the complex I enzymatic activity in two representative clones relative to total amount of complex I. We prepared MT protein extracts from mixed-age populations of WF6 short-lived clone and lake XVI-11 long-lived clone. We separated the individual ETC complexes by Blue Native polyacrylamide gel and measured the enzymatic activity of complex I using in-gel activity assay using NADH as the complex I substrate^37^. If complex one activity is intact, it shows as a purplish dark blue band indicating active complex I (Fig. 2 and Supplementary Fig. 1). Such in-gel assay is used to measure complex I enzymatic activity as the complex remains catalytically active in native gels. As seen in Fig. 2A, (lanes 4–6), the short-lived WF6 clone showed significantly less complex I enzymatic activity when compared to the long-lived XVI-11 clone (lanes 1–3). The band intensities were measured and normalized to the band intensities of total amount of complex I (blue native panels) and are shown in Fig. 2 B. The short-lived WF6 showed about 59% reduction in complex I activity compared to XVI-11. Although additional sequence variations in other components of complex I that we have not investigated here also may contribute to the reduced enzymatic activity, these results show a similar association of complex I activity to the corresponding lifespans based on studies in other model systems.

### Differences in the level of oxidative damage to proteins in the short-lived and long-lived clones

**Figure 3:**
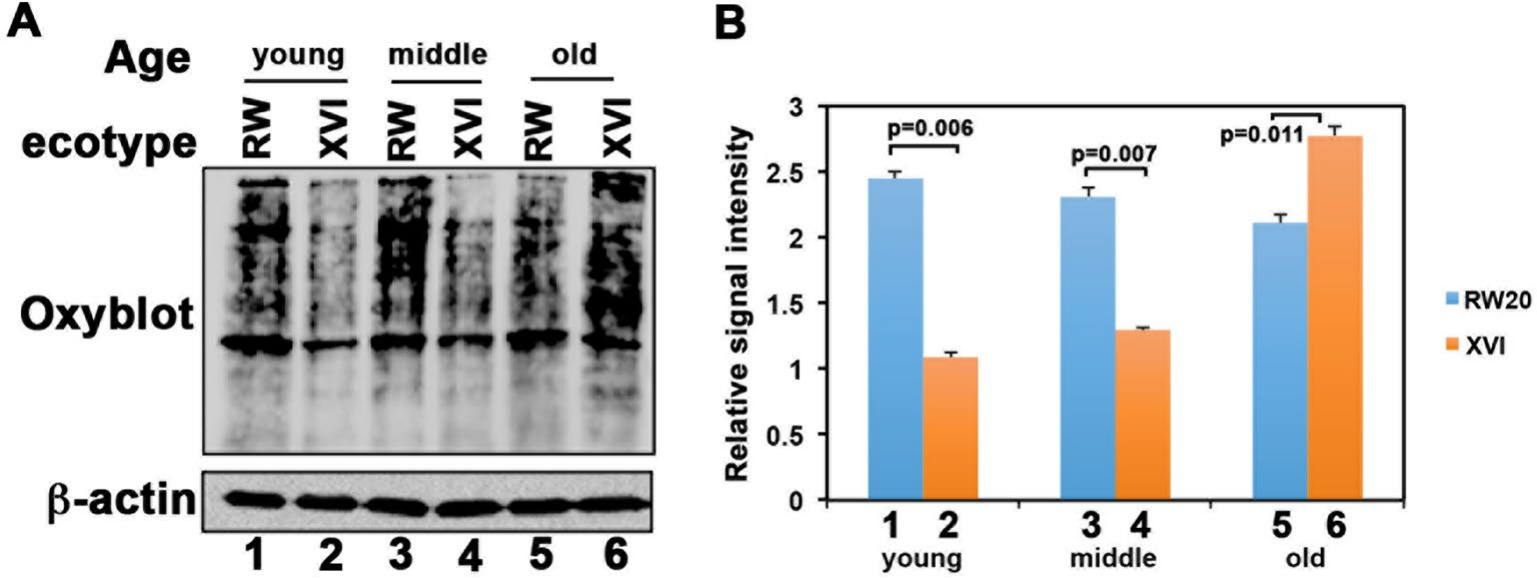
Oxidative damage to cellular proteins. (**A**) Protein carbonyl levels were measured with the Oxyblot kit (Millipore). Using total cellular extracts, the carbonyl groups in the protein side chains were derivatized to 2,4-dinitrophenyl hydrazine (DNP). Western blot analysis was performed with an antibody against DNP. Equal loading was assessed using an anti-β actin antibody (Sigma). Y: young (1 wk for both clones), M: middle aged (2 wk for short-lived RW20 and 4 wk for long-lived XVI-11) and O: old age (3 wk for short-lived RW20 and 8 wk for long-lived XVI-11). (**B**) Bar graph represents the signals obtained from the analysis of the average of 3 blots from various samples after normalization to the β-actin bands. Blue corresponds to short-lived RW20 and orange corresponds to the long-lived XVI-11.

**Figure 4:**
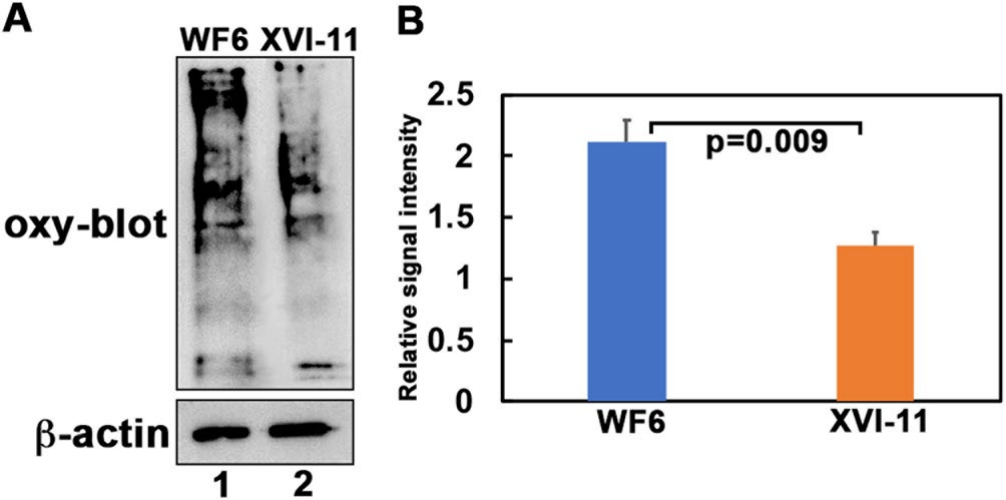
Oxidative damage to MT proteins. (**A**) Protein carbonyl levels were measured with the Oxyblot kit (Millipore). Using mitochondrial protein extracts, the carbonyl groups in the protein side chains were derivatized to 2,4-dinitrophenyl hydrazine (DNP). Western blot analysis was performed with an antibody against DNP. Equal loading was assessed using an anti-β actin antibody (Sigma). Lane 1: MT extract from mixed age populations of WF6 with about equal individuals from ages 1 wk-3 wk of the short-lived WF6 and lane 2: MT extract from mixed age populations of XVI-11 with about equal individuals from ages 1 wk-3 wk of the long-lived XVI-11. (**B**) Bar graph represents the signals obtained from the analysis of the average of 3 blots after normalization to the β-actin bands. Blue corresponds to short-lived WF6 and orange corresponds to the long-lived XVI-11.

To test if the short-lived clone generated more oxidative stress in cells, we compared the levels of oxidative protein damage at different ages in two representative clones. We performed the analysis with extracts from 1, 2, and 3 week-old organisms of the short-lived clone and 1, 4, and 8 week-old organisms of the long-lived clone which were previously determined to correspond to equivalent time points in their respective life spans^38^. As shown in Fig. 3 and Supplementary Fig. 2, the short-lived RW20 clone showed high levels of oxidative damage at a young (Y) age of 1 wk (lane 1) that remained high throughout life (lanes 3 and 5, M and O). In contrast, the long-lived clone lake XVI-11 showed dramatically less oxidative damage at a young (Y) age (lane 2) as well as during middle (M) age (lane 4) and the old (O) population showed oxidized protein levels (lane 6) that were comparable to the 1 wk-old RW20 clone (lane 1). Similarly, it can be seen in Fig. 4 and Supplementary Fig. 3 that MT extract from a mixed population of short-lived WF6 clone showed more oxidative damage to proteins, when compared to the MT extract from a mixed population of long-lived XVI-11 clone. These results demonstrate that not only does the short-lived clone and long-lived clone have variation in their MTDNA encoded ND5 protein, these differences could be playing a role in why there is a big difference in their lifespans. As the short-lived clone WF6 had less complex I enzymatic activity (Fig. 2) which is known to generate higher ROS accumulation, it would result in increased oxidative damage in both the cells as well as in the mitochondria.

### Differences in the ROS scavenger mechanisms in short- and long-lived clones

**Figure 5:**
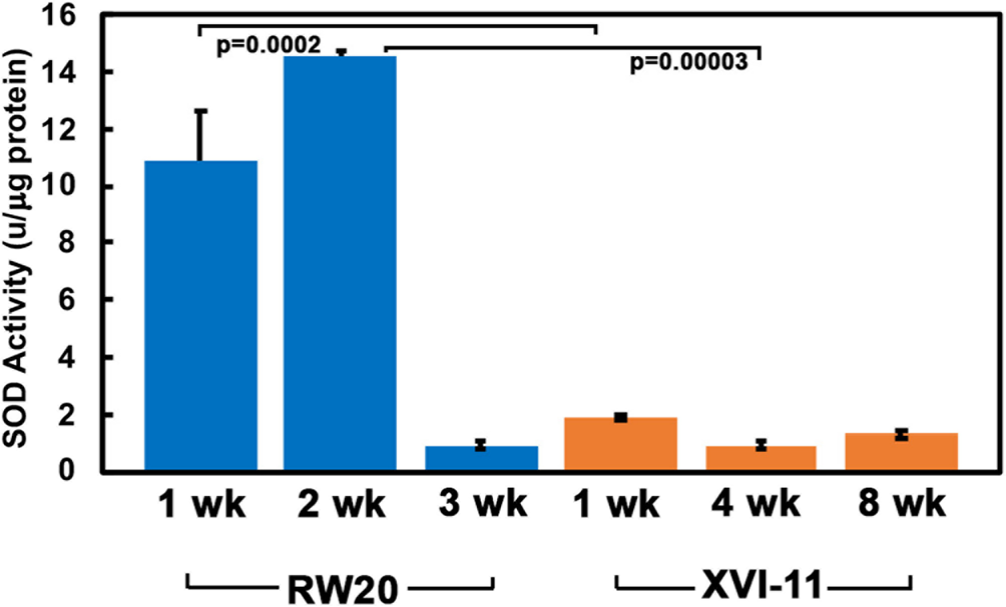
Comparison of SOD activity levels in short-lived RW20 and long-lived XVI-11. SOD activity was determined using a colorimetric assay kit (Sigma). The kit was first standardized for use with *Daphnia* samples. All reactions were performed in a 96 well plate and the activity was read on a BioTek Plate reader at a wavelength of 450 nm using the Gen5 software that was included as part of the Bio-Tek plate reader. Note that commercially available SOD was used in the assay to develop a standard curve for SOD activity. Blue bars: SOD activity at indicated ages for short-lived RW20 and orange bars: SOD activity at indicated ages for long-lived XVI-11. Error bars indicate the standard error of the mean from 4 experiments. Student T-tests were performed and the p values are as indicated.

In order to investigate additional factors that could be contributing to the difference in oxidative damage to proteins, we compared the activity of the ROS scavenger enzymes superoxide dismutase (SOD) and catalase (CAT) in the short- and long-lived clones. SOD and CAT are ROS-scavenging enzymes known to play a central role in the antioxidant defense systems in all organisms^39^. SOD enzymes convert the toxic superoxide radical into hydrogen peroxide (another ROS) and molecular oxygen depending on the cellular context^40,41^. As seen in Fig. 5, the short-lived RW-20 displays significantly higher levels of SOD activity at young and middle ages, 10.9 and 14.5 units per microgram of protein respectively, whereas the long-lived clone XVI-11 at equivalent ages shows only 1.9 and 0.9 units per microgram of protein respectively. At old age in RW20*,* the SOD activity declines drastically to levels similar to old XVI-11 *with* 1.3 and 0.9 units per microgram of protein, respectively. Of particular note, XVI-11 exhibits relatively low amounts of SOD activity at all ages examined with very little change through the lifespan indicating lower amounts of ROS accumulation as ROS production is known to induce expression of SOD.

**Figure 6:**
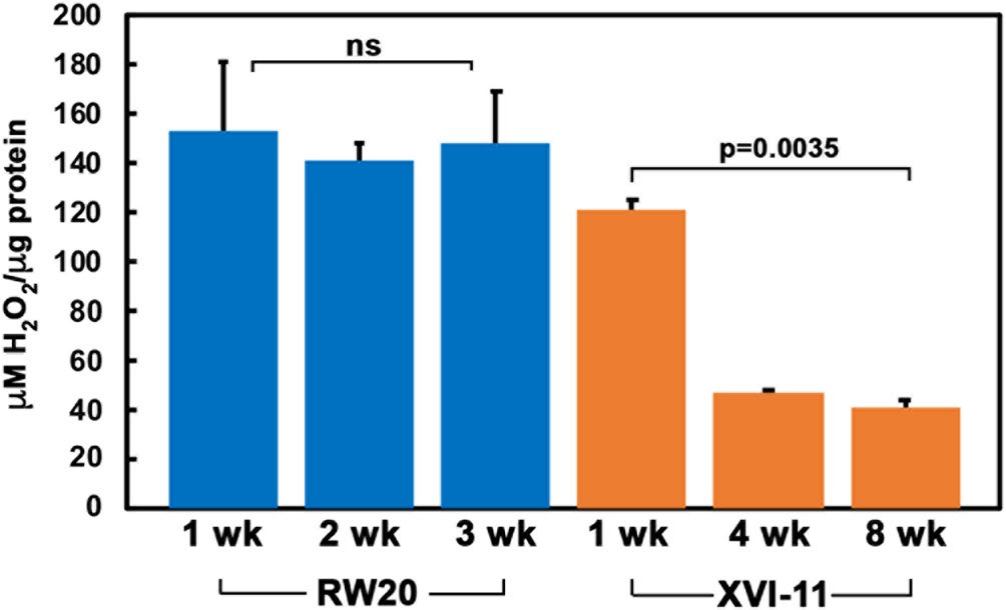
Comparison of hydrogen peroxide levels in short-lived RW20 and long-lived XVI-11. Hydrogen peroxide levels were determined at the indicated ages for short-lived RW20 and using a kit available through Pierce. Before the kit, which uses a colorimetric assay, was used with samples from aged *Daphnia*, the kit was standardized for use with *Daphnia* samples (Data not shown). All reactions were performed in a 96 well plate that was read on a BioTek Plate reader at a wavelength of 560 nm using the Gen5 software that was included as part of the Bio-Tek plate reader. Blue corresponds to short-lived RW20 and orange corresponds to the long-lived XVI-11. Error bars indicate the standard error of the mean from 3 experiments. Student T-tests were performed and the p values are as indicated, ns means not significant.

As the short-lived RW20 showed high level of oxidative damage to proteins at all ages during lifespan (Figs. 3 and 4) and also had elevated levels SOD activity (Fig. 5), we next examined the relative levels of hydrogen peroxide, the SOD scavenging reaction product that is also a ROS, in both short- and long-lived clones. Hydrogen peroxide causes oxidative damage in macromolecules including proteins^40,41^ and eleven distinct mitochondrial sites have been identified that can produce it in addition to the SOD enzyme reaction. As is shown in Fig. 6, the short-lived RW20 contains relatively high levels of hydrogen peroxide with little change during the lifespan. The young, middle and old RW20 organisms showing 153.6, 139.7, and 147.9 µM hydrogen peroxide per microgram protein respectively. In contrast, the long-lived XVI-11 *clone* contains high levels of hydrogen peroxide only in young organisms with 139.3 µM hydrogen peroxide per microgram protein, which declines drastically by middle age and is kept relatively low even in old organisms at 46.5 and 41.1 µM hydrogen peroxide per microgram protein respectively.

**Figure 7:**
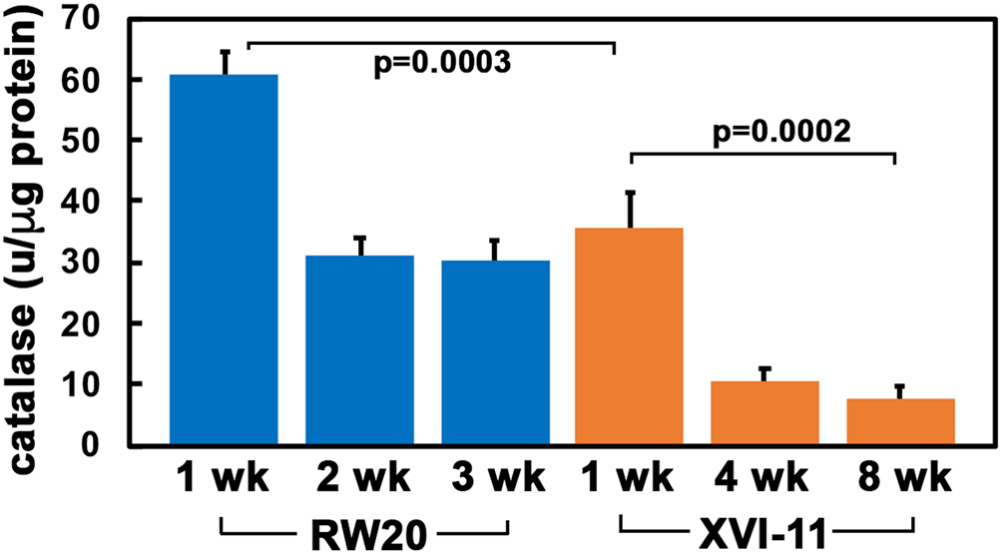
Comparison of catalase activity in RW20 and XVI-11. Catalase activity was determined using a previously published protocol based upon the extinction of hydrogen peroxide over a period of time. All reactions were performed in a 96 well plate that was read on a BioTek Plate reader at a wavelength of 240 nm using the Gen5 software that was included as part of the Bio-Tek plate reader. Plates were read every 47 s for a duration of 5 min and the extinction of hydrogen peroxide was calculated. The activity of catalase was then calculated based upon the slope of the line representing the disappearance of hydrogen peroxide. Blue corresponds to short-lived RW20 and orange corresponds to the long-lived XVI-11. Error bars indicate the standard error of the mean from 3 experiments and the p-values are as indicated.

We then investigated the activity of catalase, another ROS scavenger enzyme that enzymatically converts the toxic hydrogen peroxide to water and molecular oxygen^1,40,41^. To analyze the activity of catalase, we used a catalase activity assay as previously described^42,43^. We initially standardized the assay using commercially available catalase (Sigma) and also optimized the assay parameters for *Daphnia* samples. As is shown in Fig. 7, young RW20 exhibit the highest catalase activity with an average of 63.4 units of CAT per microgram of protein with a decline to 30.9 units per microgram of protein at middle age and thereafter staying at 30.4 units of CAT per microgram of protein in older age. In XVI-11 the same overall trend is observed with higher amounts of catalase being in young organisms before declining in middle age and staying relatively unchanged in old organisms (44.6 to 10.4 to 7.7 units of CAT per microgram of protein, respectively. However, the amount of catalase activity is significantly lower in XVI-11 at equivalent time points compared to RW20 and as ROS is known to induce catalase expression, this possibly means lower overall levels of ROS in XVI-11.

## Discussion

Multiple studies from several model organisms reveal a complex relationship between the harmful ROS and aging. The role of oxidative damage and the effects of ROS on the aging process is not as straightforward as originally envisaged. Initial studies performed in *D. melanogaster* depicted ROS as being quite detrimental to organisms and was cited as being a major factor for the aging process^44–46^. Later on, other studies indicated that high ROS levels do not necessarily cause shorter lifespan and that overexpression of certain scavenger enzymes known to mitigate effects of ROS showed no effect on longevity^1,45–47^. One theory poses the presence of ROS as necessary for survival and can be thought of as molecules similar to AMP and NAD+, molecules that are necessary in a strict physiological range and are essential for proper homeostasis^1^. It is plausible that when ROS levels are elevated beyond this physiological range, they then become detrimental to the organism by causing damage to macromolecules, with the most crippling damage being to the proteins. ROS acts as a stress response signaling cascade that is not initially causative of aging and several ROS, particularly hydrogen peroxide, have been shown to be essential signaling molecules^1,48^.

In our present study we sought to examine the overall amount of oxidative damage to proteins, the activity levels of several ROS scavenger enzymes, and an associated ROS concentration level in young, middle, and old organisms of closely related *Daphnia* with vastly different lifespans, RW20 or WF6 for short-lived and XVI-11 for long-lived. Some of the previous studies have demonstrated DNA and lipid damage from ROS aren’t likely involved as large causative agents for the aging process^45,49^. Therefore, we decided to examine oxidative damage in proteins and based on our data, WF6 and RW20 exhibit more oxidative damage to proteins than XVI-11 at young and middle-ages but the amount of damage is comparable in old organisms of both clones. What is causing the difference in oxidative damage to proteins? Based on our results, the naturally occurring ND5 sequence variations observed in short-lived RW20 and WF6 correspond to reduced complex I activity, which is known to lead to higher ROS generation. Thus, the short-lived clones generate more ROS and that corresponds well with higher amounts of oxidative damage to both cytoplasmic and mitochondrial proteins. Thus, we are able to associate the natural variations in mitochondrial ND5 sequence to lower complex I activity and increased oxidative damage to proteins in *Daphnia* clones with shorter lifespans.

The ROS scavenger enzyme SOD activity levels were much higher in the short-lived RW20 than long-lived XVI-11. This enzyme is known to mitigate the extremely toxic free radical superoxide anion; however, the product of the enzymatic reaction that rids the cells of these toxic species is hydrogen peroxide which is another toxic ROS. Therefore, the high amount of SOD activity in RW20 may not be beneficial for the organism as the enzyme produces a toxic product that could further damage cellular macromolecules, especially proteins. The hydrogen peroxide species is much more stable than superoxide anion and could have a lasting detrimental effect on various macromolecules and can be converted into another toxic ROS, the hydroxyl radical^48^. We also analyzed the levels of hydrogen peroxide in short- and long-lived clones at young middle and old ages. The short-lived RW20 did in fact have higher levels of hydrogen peroxide levels than XVI-11 and these higher levels are maintained throughout life. Note that SOD activity diminishes in old age of RW20 but the hydrogen peroxide levels remain high. As mitochondrial respiration could be less efficient with age and more ROS is known to be produced from aberrant ETC reactions, that could be the cause of the high level of hydrogen peroxide in old RW20^1,50^. The long-lived XVI-11 also displayed higher amounts of hydrogen peroxide in young organisms which diminished by middle age and was maintained low through old age. To follow this up, we examined CAT activity, the enzyme which would convert hydrogen peroxide into water and molecular oxygen and thus can potentially alleviate some of the ROS induced stress in the cells. The results indicated that although RW20 showed higher amounts of catalase activity there was much higher amounts of hydrogen peroxide as well. This explains why, the short-lived RW20 contains higher amounts of oxidative damage to proteins, being unable to cope with the ROS produced during normal metabolism throughout the lifespan.

It’s also important to note that there are other types of ROS and ROS scavenging enzymes that we did not assay in this study, including hydroxyl anions and glutathione peroxidases^51–54^. Thus, whether free radicals and ROS are causative of aging, or act as a signaling molecules in response to other aging related insults, we definitely can detect differences in MT complex I activity, ROS levels, ROS scavenging enzymes, and oxidative damage to proteins between the short- and long-lived clones of *D. pulex.* In order to evaluate the direct causal relationship between lifespan and naturally occurring sequence variations in ND5 and possibly other MT genes as well as nuclear genomes, various mitochondrial genomes would need to be manipulated on different nuclear backgrounds and can be explored in future studies.

Crosstalk between the ROS network and the heat shock response has been observed and is important for aging process and longevity^55–57^. In a previous study, we had examined the proteotoxic response known as the heat shock response in short- and long-lived clones and found that the short-lived clone stops responding to heat stress by middle age whereas the long-lived clone can still respond to heat shock in middle age by inducing the expression of heat shock protein 70, a molecular chaperone^38^. Thus, the ability to respond to proteotoxic heat stress varies between the short- and long-lived clones of *Daphnia* and may also contribute to the observed differences in the longevity. Other ROS not analyzed in this work, such as the hydroxyl ions and their associated scavenging enzymes can be analyzed in future to determine their role in oxidative damage and lifespan. More importantly, we could further analyze the effects of exogenous treatment with antioxidants on lifespan extension, rate of aging, or sex-dependent aging^58–60^ using *Daphnia* model system.

Our work provides an entry point for the joint consideration of proximate and ultimate causes of differences in lifespan and aging. Ultimate causes are evolutionary, and in our system genetic differences have been shown to be associated with ecological differences in selection pressures on aging^28,30,61,62^. Our ecotypes, whether they are considered sister species that continue to exchange genes, incipient species in the throes of speciation, or simply divergent populations of one larger species, have strong differences of both life history and mitochondrial function. While the differences in mitochondrial function almost certainly are proximate contributors to differences of life span and aging, they are unlikely to be the only proximate causes. We cannot tell whether the mitochondrial functions we examined were the initial molecular mechanisms underlying the phenotypic differentiation of the ecotypes, because as evolutionary change in aging occurs, feedback loops are expected to change selection pressures on other proximate mechanisms that contribute to aging (e.g., differences in heat shock response). Whether the differences we found are limited to our specific clones or are broadly representative of the adaptive divergence of the ecotypes is uncertain without broader sampling. However, it is worth noting that sequencing ND5 is often used as a shortcut for taxonomic identification because the ecotypes are morphologically indistinguishable^32,63,64^, and thus we expect that functional differences driven by ND5 will be linked to taxonomic identifications made with ND5.

It is also important to keep in mind the limitations of our study. One of the limitations of our study is that any trait or gene that differs between the chosen short- and long-lived ecotypes will possibly score as associated with differences in lifetime and aging. Nevertheless, it is interesting that genes and molecular processes that have previously been identified as possible proximate drivers of lifespan and aging in other model organisms differ between these *Daphnia* ecotypes as well. In order to establish a direct link between naturally occurring mitochondrial genome variations with aging and lifespan, various mitochondrial genomes will need to be combined with different nuclear backgrounds of short -and long-lived ecotypes. This is certainly possible by setting up genetic crosses, as *Daphnia* can reproduce sexually and parthenogenetically allowing isogenic organisms to be easily produced. Our studies indicate that *Daphnia* would be the best suited model organism for examining and establishing the role of natural variations in mitochondrial genomes, oxidative damage to proteins, in rates of aging and relative lifespans.

## Methods

### Daphnia cultures

*Daphnia* clones used in this study were isolated from ponds and lakes in southwest Michigan in 2004–2008 and have since been cultured in the lab. No specific permissions are required to collect zooplankton from these public-access waterbodies in Michigan. *D. pulex* clones are neither endangered nor protected. For further details on the source populations, see Dudycha^30^. *Daphnia* are maintained at a temperature of 20 °C with a photoperiod of 12:12 L:D (12 h of light followed by 12 h of dark) within a Percival growth chamber. *Daphnia* were maintained at a concentration of 3 to 5 adult animals per 250 ml beaker in 200 ml of COMBO, an artificial lake water^65^. *Daphnia* were cleared of young and transferred to a new beaker with fresh water on thrice weekly. They were fed every day with vitamin- supplemented algae *Ankistrodesmus falcatus* at a concentration of 20,000 cells/ml^66^. To generate experimental animals, even-aged cohorts were begun by placing neonates individually in 100 ml of COMBO. Experimental animals were otherwise maintained as in the source cultures.

### Survivorship curves

We compared survivorship of the 11 different clones using previously described methods for *Daphnia* in^60^. Experimental conditions were 20 °C, a 12:12 L:D photoperiod, with animals kept in individual 150-ml Pyrex beakers in 100 ml COMBO hardwater artificial lake water^65^. Two generations were maintained under experimental conditions prior to initiating the life table to minimize maternal effects variation. Experimental individuals were fed 20,000 cells/ml *Ankistrodesmus falcatus* daily, a food level that produces normal aging processes in *Daphnia*^28^. Individuals were transferred to fresh beakers and COMBO thrice weekly, and survivorship was recorded at each transfer until all experimental animals died. For each clone, n = 60 females, divided into two blocks of 30 individuals per clone per block. For survival curves, data were pooled across blocks. Individuals that died as juveniles (before age = 9 days, 47 individuals total across all clones and blocks) were excluded from analysis.

### ND5 sequence analysis

#### Cloning of *Daphnia* ND5 ORF

Primers were designed to PCR amplify the *Daphnia* ND5 open reading frame (ORF) sequences of all our long- and short-lived *Daphnia* clones using MacVector based on the published *D. pulex* genome on the wfleabase.org. For each PCR reaction, 10 ng genomic DNA was used with 50 pmol each of the forward and reverse primers designed to amplify the ND5 PCR product using the Promega GoTaq PCR kit. The following conditions were used for PCR: Lid 95 °C, 95 °C for 2 min (initial denaturation), denaturation at 95 °C for 15 s, annealing at 55 °C for 15 s, extension at 72 °C for 15 s for 24 cycles on the Eppendorf Scientific gradient Mastercycler®. PCR products were separated on a 1% agarose gel. The PCR products were sent out for sequencing (Eton Bio). Both DNA sequences and the deduced protein sequences were aligned using MacVector and compared to the reference *Daphnia* mitochondrial genome. The protein sequences were checked for any amino acid sequence changes.

Primer sequences used were as follows:

- Primer: ND5 Forward: 5’-GAGGTGGTCCGCATTCTTTA-3’.
- Primer :ND5 Reverse: 5’-AAAGTCAAGTAGCGCGGGTA-3.

#### *Daphnia* cell lysate isolation preparation for Blue Native gels

*Daphnia* were collected from ongoing cultures into 1.5 ml microcentrifuge tubes. All water was removed from the tubes containing the *Daphnia*, the organisms were washed once with 1 ml cold PBS and the organisms were homogenized using a tight-fitting pestle. We homogenized 100–200 individuals in 100 µl of NativePAGE® Sample buffer, 5% Digitonin containing protease inhibitor cocktails (Sigma and Calbiochem) following the manufactures protocol for the NativePAGE® Sample Prep Kit (BN206, BN 2008, Life Technologies) and then centrifuged at 20,000×*g* for 30 min at 4 °C. The supernatant was aliquoted into sterile microcentrifuge tubes and stored at − 80 °C until use. The protein concentration was determined using a BCA kit (Pierce). To determine the activity of mitochondrial complex I in the two ecotypes of *Daphnia* at different ages, we utilized the blue native gel kit from Life Technologies (NativePAGE™ Novex® Bis–Tris Gel system, BN1001BOX). After mitochondrial proteins were extracted, two Gels were run side by side; (1) a clear native gel used for the in-gel activity assay and (2) a blue native gel for staining with Coomassie blue G250.

#### Blue Native (BN)-polyacrylamide gel electrophoresis (PAGE)

For BN-PAGE we used protocol as explained by Jha et al.^67^. Mitochondrial protein extracts prepared in 1X Native PAGE™ Sample Buffer and detergents were subsequently run on a 4–16% Blue Native polyacrylamide gradient gel following the manufacturer’s protocol (Invitrogen, Native PAGE Gel system). Immediately prior to electrophoresis, NativePAGE™ 5% G-250 Sample Additive was added to the samples and the NativeMark™ Unstained Protein Standard (Invitrogen, LC0725) was used as the molecular weight markers. Two types of NativePAGE™ Running Buffers are used for native gel electrophoresis with NativePAGE™ Novex® Bis–Tris Gels. NativePAGE™ 20X Running Buffer (BN2001) and the NativePAGE™ 20X Cathode Buffer Additive (BN2002), contains 0.4% Coomassie G-250 (added to NativePAGE™ Running Buffer to generate the Cathode Buffer) which were purchased and prepared according to the manufactures protocol. The dark cathode buffer (Dark Blue Cathode Buffer contains 0.02% G-250) was used for the Blue native gel. The manufacturer’s instructions for performing electrophoresis using the Invitrogen Mini-Gel Tank (Invitrogen, A25977) was followed with the gel being run in a cold room at 4 °C. The gel was run first at 150 V for 60 min, then at 250 V for the remainder of the run (30–90 min). After electrophoresis was completed, a fast Coomassie G-20 staining was performed by first placing the gel in 100 ml Fix solution (40% methanol, 10% acetic acid) and microwaving on high (950–1100 watts) for 45 s. The gel was then placed on an orbital shaker for 15 min at room temperature. Finally, to destain the gel, we added 100 ml destain solution (8% acetic acid) and microwaved on high (950–1100 watts) 45 s. The final round of shaking was done on an orbital shaker at room temperature until the desired background was obtained. The gel was then washed with water and photographed using a White Light Transilluminator FB-WLT-417 (Fisher Scientific).

#### In-gel complex I activity assays using Clear Native (CN)-PAGE

The protocol used for the BN-PAGE was also used for the CN-PAGE, with the major changes being the use of a light Cathode buffer (made by adding less NativePAGE™ Cathode Buffer Additive (20X) to the NativePAGE™ Running Buffer). The gel was first run in the light cathode buffer at 150 V for 30 min. After 30 min, the light cathode buffer was changed to the clear cathode/running buffer to prevent the excessive blue color of the Coomassie Blue dye from interfering with the color of the in-gel activity assay. The gel was then incubated for 30 min in 20 ml of the Complex I activity substrate buffer (2 mM Tris–HCL at pH 7.4, 0.1 mg/ml NADH disodium salt trihydrate (Abcam, ab146315 and 2.5 mg nitrotetrazolium blue (NTB) (Abcam, ab146262). To stop the reaction once the desired purple color for complex I activity was obtained to fix the gel, a 10% acetic acid solution was used. The gel was then washed with water and photographed using a White Light Transilluminator FB-WLT-417 (Fisher Scientific).

To determine the relative activity of complex I to the total amount of complex I protein, the band intensities on the BN gel and CN gel were measured and the relative activity at the three protein concentrations were calculated as relative complex I activity = band intensity for the in-gel activity assay/band intensity on the BN gel for 15, 30, and 40 μg lanes.

### Detection of protein carbonyls via Oxyblot

The oxidation status of proteins from *Daphnia* clones was determined using the Oxyblot protocol from EMD Millipore. This assay is based upon the fact that reactive oxygen species induce carbonyl group formation in proteins at specific residues. These carbonyl groups can then be derivatized into 2,4-dinitrophenylhydrazone (DNP-hydrazone) by 2,4-dinitrophenylhydrazine (DNPH). The altered groups are detected following SDS PAGE and Western Blot Analysis with an antibody that detects the DNP-hydrazone group of oxidized proteins. *Daphnia* protein samples were harvested using RIPA buffer and protein concentration was determined using a BCA protein quantification kit (thirty individual *Daphnia* were used for each aged set of both ecotypes). To derivatize the carbonyl group into DNP-hydrazone, 20 µg of each protein sample was placed in a fresh tube (the samples were diluted such that 5 µl of each sample was used). Then 5 µl of 12% SDS was added to the tube to denature the proteins and 10 µl of 1X DNPH solution was added to the tube. The tubes were then incubated for 15 min at room temperature. The derivation reaction was stopped by adding 7.5 µl of neutralization solution to each tube. Note that we performed each Oxyblot with duplicate samples, one that had been derivatized and a control that was not derivatized into DNP-hydrazone. The 20 µg samples were loaded onto a 12% polyacrylamide gel and western blot analysis was performed using chemiluminescence ECL plus for detection of protein on a GE Typhoon LAS 4000 (antibody used was provided with the Oxyblot kit, recognizes DNP-hydrazone, dilution 1:2000)^38^. The images were analyzed and quantified using the program ImageQuant.

### Hydrogen peroxide assay

Hydrogen peroxide amounts were determined for each aged set of *Daphnia* using the Pierce Quantitative Peroxide Assay Kit (Lipid). This assay is based upon the oxidation of ferrous iron to ferric iron which is performed directly by the hydrogen peroxide contained within the sample. Once the iron is in the ferric state, it can bind directly to a xylenol orange dye which produces a violet color that can be measured spectrophotometrically at a wavelength of 560 nm. We first standardized the assay using various amounts of hydrogen peroxide (30% w/v) and then performed the assay with *Daphnia* samples. Ten individual *Daphnia* were homogenized in 100 µl of PBS without any added protease inhibitors and centrifuged at 13,200 rpms for 10 min. The supernatant was placed in a new microcentrifuge tube and used for the assay. Note for each experiment 3 biological replicates and 3 technical replicates were performed. To perform the assay, we added 20 µl of sample with 200 µl of the working reagent supplied with the kit in a 96 well plate. The plate was incubated at room temperature for 20 min before being read on a plate reader (Bio-Tek) using Gen5 software that was included as part of the Bio-Tek plate reader. Note that all concentrations of hydrogen peroxide were normalized to one microgram of protein which was determined using a BCA kit from Pierce.

### Catalase activity assay

Catalase activity was detected using a modified method first developed by Beer and Sizer^42,43^. This spectrophotometric method utilizes the fact that the absorbance of hydrogen peroxide can be detected at 240 nm. The higher the amount of catalase present, the faster the absorbance by hydrogen peroxide will be diminished^42^. Seven individual *Daphnia* from each ecotype at the 3 different ages were homogenized in 30 µl of 67 mM Phosphate Buffer Saline (PBS) with protease inhibitor cocktail (Sigma-Aldrich, P8340). Note for each experiment 3 biological replicates and 3 technical replicates were performed. The homogenate was centrifuged at 13,200 rpms for 5 min and the cleared supernatant was kept and used in the Catalase Activity Assay. The assay was initiated by adding 300 µl of PBS-hydrogen peroxide solution (comprised of 160 µl of 30% hydrogen peroxide and 99.84 ml of 67 mM PBS) into a clear bottom well 96 well black plate (UV-star plate from Greiner Bio-one). Then 5 µl of *Daphnia* homogenate was added to the PBS-hydrogen peroxide solution in the well, mixed and read on a 96 well plate reader (Bio-Tek) using Gen5 software that was included as part of the Bio-Tek plate reader. Numerous reads were taken over a duration of 5 min with reads occurring every 47 s. We then calculated the slope of the diminishing hydrogen peroxide amounts and calculated the activity of catalase based upon the following equation where S is equal to the slope describing the diminishing hydrogen peroxide absorbance, V is volume of PBS-hydrogen peroxide solution added per well, and ε is the molar extinction rate of hydrogen peroxide at 240 nm (0.0436 ml µmol^−1^ cm^−1^ at 240 nm)^43^.

$$Catalase\ Activity=S\left(\frac{V}{\varepsilon }\right)$$

For each aged *Daphnia* set, we also determined the protein content of the samples using BCA kit from Pierce and present the data as units of catalase per microgram of protein. Note we also performed standard calculations based upon commercial catalase isolated from bovine liver (Sigma). For the standard calculation, we serially diluted the catalase and performed the assay at various concentrations to standardize the assay.

### Superoxide dismutase (SOD) activity assay

To determine the activity of SOD in the two ecotypes of *Daphnia* at different ages, we utilized a kit from Sigma (Sigma SOD Assay kit 19160). This kit is based upon an altered tetrazolium salt WST-1, which will be reduced by superoxide anion to form a formazan dye which can then be detected spectrophotometrically at 450 nm wavelength. The presence of SOD will reduce the amount of superoxide anion and thus diminishing the amount of dye produced lowering the absorbance detected at 450 nm. For each aged set of *Daphnia*, we homogenized 10 individuals in 100 µl of PBS with protease inhibitor cocktail (Sigma-Aldrich, P8340), and then centrifuged at 13,200 rpms for 10 min. The supernatant was kept and used as the samples for the SOD Activity Assay. Note for each experiment 3 biological replicates and 3 technical replicates were performed. To perform the assay, 20 µl of *Daphnia* homogenate was added to the well of a 96 well plate. Then 200 µl of WST working solution was added to the well and mixed followed by the addition of 20 µl of Enzyme Working Solution. The plate was then incubated for 20 min at 37 °C before being read using a plate reader (Bio-Tek) at 450 nm using Gen5 software that was included as part of the Bio-Tek plate reader. The amount of SOD needed to inhibit 50% of the reduction of the WST-1 dye is defined as one unit of SOD^43^. Note that the amount of protein was determined for each sample and the activity of SOD is reported as units of SOD per microgram of protein. To standardize the assay we also performed the experiment using varying amount of commercially available SOD (sigma).

### Statistics

To determine statistical significance when analyzing the various assays performed in this study, we executed both a two tailed Student’s T-test, assuming equal variance as well as Nonparametric Wilcoxon test. Each figure legend denotes p values, note that our alpha level was p = 0.05. Importantly, the p-values from both approaches agreed well in every single case.

## Supplementary Information

Supplementary Figures.

## Data Availability

All data generated during this study are included in this article.

## Abbreviations

ROS: Reactive oxygen species
MT: Mitochondrial
ETC: Electron transport chain
ND5: NADH dehydrogenase subunit 5
mtDNA: Mitochondrial DNA
LHON: Leber's hereditary optic neuropathy
MELAS: Mitochondrial encephalomyopathy, lactic acidosis, and stroke-like episodes
ORF: Open reading frame
BN: Blue Native
CN: Clear Native
SOD: Superoxide dismutase
CAT: Catalase
