# Supplementary material for: Relationship between oxidative stress and lifespan in Daphnia pulex
Source: Sci Rep. 2022 Feb 11;12:2354. doi: 10.1038/s41598-022-06279-4 (PMC8837783; doi:10.1038/s41598-022-06279-4)
Supplement: Supplementary file 1 — Supplementary Figures. [file 41598_2022_6279_MOESM1_ESM.pdf]

Blue Native

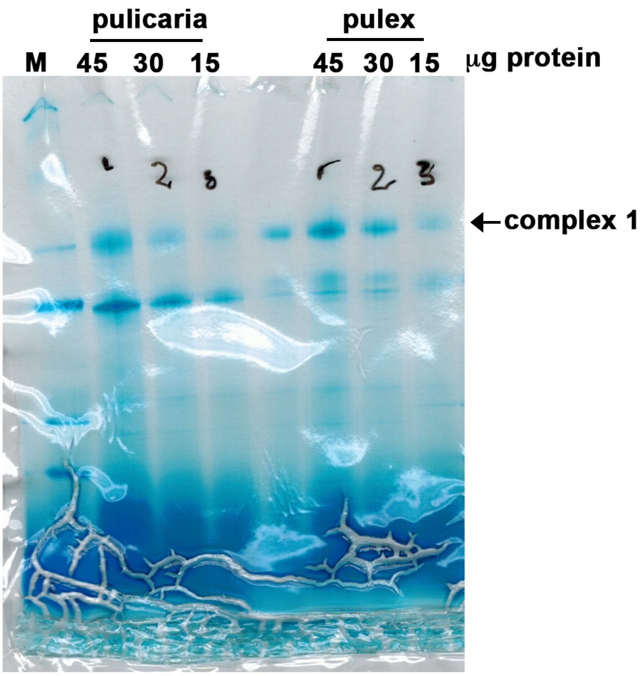

In-gel activity

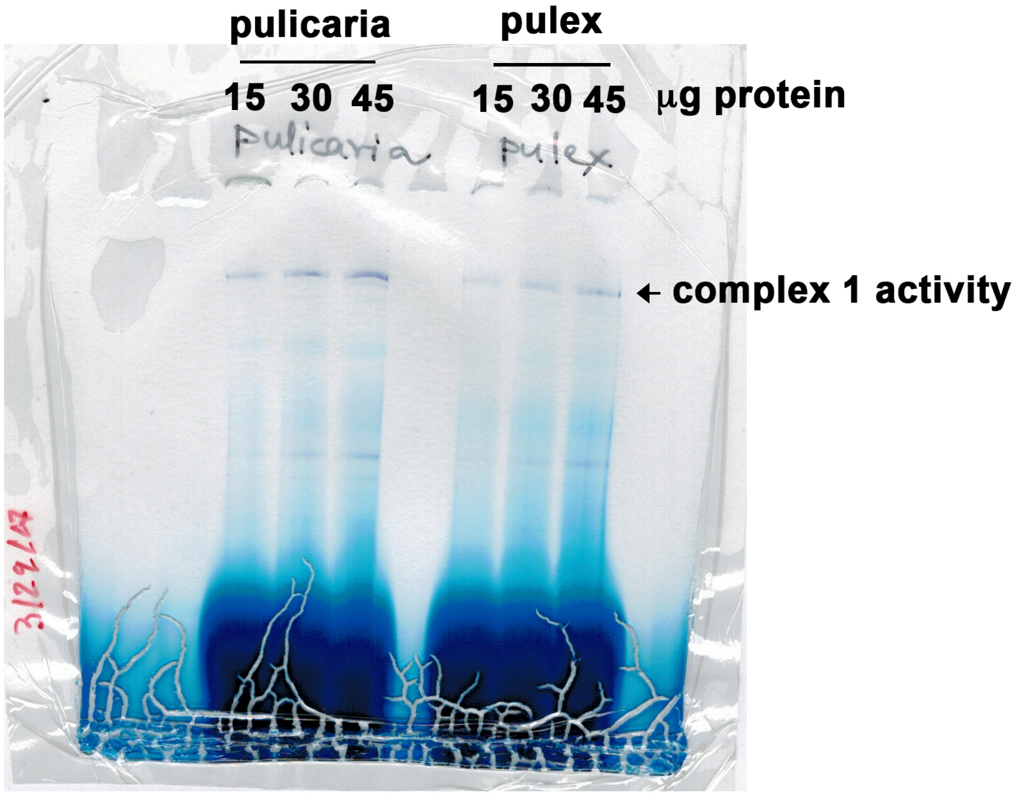

Supplementary Fig. 1

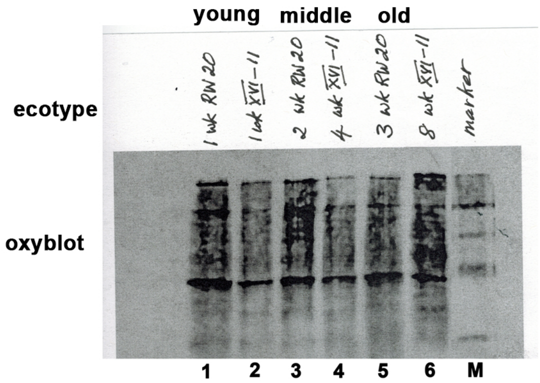

western blot for equal loading control

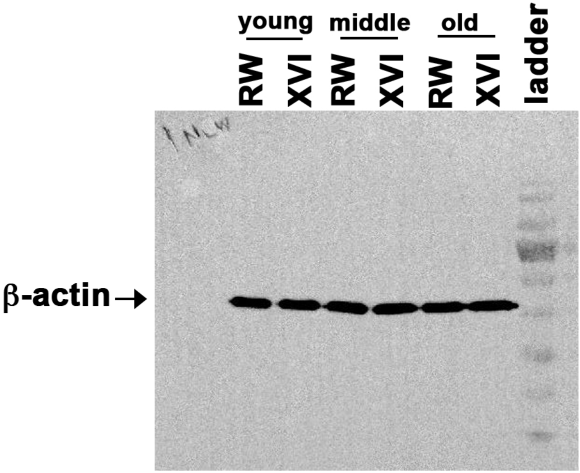

Supplementary Fig. 2

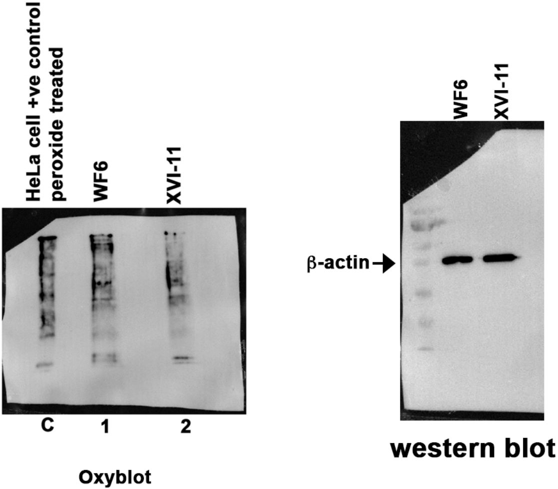

Supplementary Fig. 3

Legends for Supplementary Materials

Figure 1. Complex I activity from clones *WF6* and *XVI-11.*

Blue Native gels and in-gel activity assay for complex I. Extracts made from short-lived WF6 and long-lived XVI-11 clones were run on Blue Native gel for total amount of complex I and a duplicate gel was stained for measuring complex I activity. Lanes 1- 3: 15μg, 30μg, and 45μg of total *extract* prepared from WF6 mitochondria and lane 4-6: 15μg, 30μg, and 45μg of total extract prepared from XVI-11 mitochondria.

Figure 2. Oxidative damage to cellular proteins.

Protein carbonyl levels were measured with the Oxyblot kit (Millipore). Using total cellular extracts, the carbonyl groups in the protein side chains were derivatized to 2,4-dinitrophenyl hydrazine (DNP). Western blot analysis was performed with an antibody against DNP. Equal loading was assessed using western blot analysis with an anti-β actin antibody (Sigma). Y: young (1 wk for both clones), M: middle aged (2 wk for short-lived RW20 and 4 wk for long-lived XVI-11) and O: old age (3 wk for short-lived RW20 and 8 wk for long-lived XVI-11).

Figure 3. Oxidative damage to MT proteins.

Protein carbonyl levels were measured with the Oxyblot kit (Millipore). Using mitochondrial protein extracts, the carbonyl groups in the protein side chains were derivatized to 2,4-dinitrophenyl hydrazine (DNP). Western blot analysis was performed with an antibody against DNP. Equal loading was assessed using a western blot analysis with an anti-β actin antibody (Sigma). Lane 1: MT extract from mixed age populations of WF6 with about equal individuals from ages 1 wk-3 wk of the short-lived WF6 and lane 2: MT extract from mixed age populations of XVI-11 with about equal individuals from ages 1 wk-3 wk of the long-lived XVI-11.
